# Supplementary material for: Synthesis of New Amino-Functionalized Porphyrins:Preliminary Study of Their Organophotocatalytic Activity
Source: Molecules. 2023 Feb 20;28(4):1997. doi: 10.3390/molecules28041997 (PMC9967531; doi:10.3390/molecules28041997)
Supplement: Supplementary file 1 [file molecules-28-01997-s001.zip › molecules-2209874-supplementary-final.pdf]

Molecules
Supplementary Material:

Electronic Supporting Information for

Synthesis of New Amino-Functionalized porphyrins: Preliminary Study of Their Organophotocatalytic Activity

Pol Torres, Marian Guillén, Marc Escribà, Joaquim Crusats and Albert Moyano*

Section of Organic Chemistry, Department of Inorganic and Organic Chemistry, University of Barcelona, Faculty of Chemistry, C. de Martí i Franquès 1-11, 08028 Barcelona, Spain

SUMMARY

- Stereochemical assignation of imidazolidinone-porphyrin hybrids **6b** and **6b’** (ESI2-ESI4)
- Experimental procedures for the preparation of compounds **TPPH$_2$, Na$_4$TPPH$_2$S$_4$, (H$_3$O)$_2$TPPH$_4$S$_4$, 4a-c, 18, 25, 32, 33,** and **34** (ESI5-ESI10)
- NMR spectra of new compounds (ESI11-ESI18)
- Determination of the stereochemical composition of the products of the Diels–Alder cycloaddition of cyclopentadiene (**28**) and (*E*)-cinnamaldehyde (**29**) (ESI19-ESI20)

Stereochemical assignation of imidazolidinone-porphyrin hybrids 6b and 6b’

The condensation reaction between the copper(II) complex of 2-formyl-5,10,15,20-tetraphenylporphyrin (**3**) and the methyl amide **4b**, derived from L-phenylalanine, furnished the Cu(II)-imidazolidinone **5b** in 50% yield as an unknown mixture of diastereomers (two spots by TLC). Demetallation of **5b** with concentrated sulfuric acid, followed by neutralization and filtration over a silica gel pad with dichloromethane:methanol 98:2 afforded the demetallated imidazolidinone as a mixture of diastereomers **6b**/**6b’** in 78% global yield. Careful chromatographic purification allowed the isolation of the pure diastereomers **6b** and **6b’** in 18% and 26% yield, respectively (Scheme 3 in the main text).
The stereochemical assignment of these products could be performed by analysing the different splitting pattern observed in both diastereomers in their respective ^{1}H NMR spectrum (CDCl$_3$, 400 MHz). As shown in Figure S1, clear divergences arise in the signals belonging to the three-spin system formed by the diastereotopic benzylic protons (AB/AM) and to the α-carbonyl proton (X), between 4.00 and 2.60 ppm.

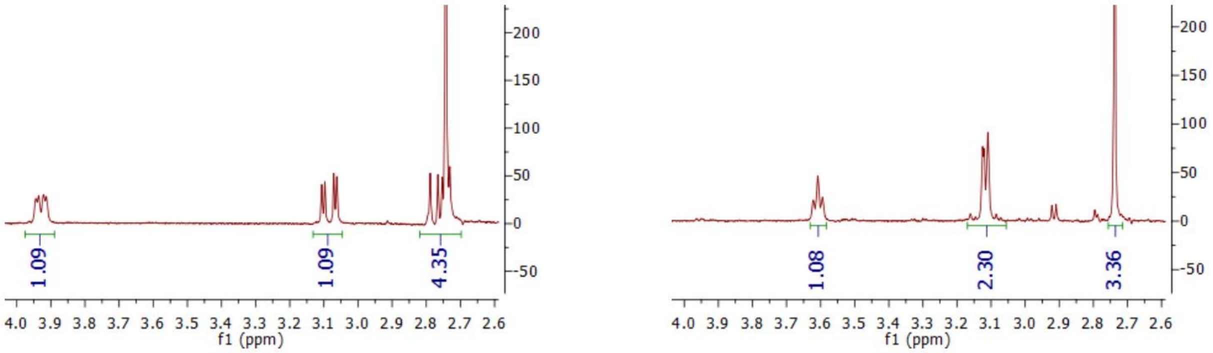

Figure S1. ^{1}H NMR spectra ($CDCl_3$, 400 MHz) of compounds **6b** (minor) and **6b'** (major) between 4.00 and 2.60 ppm.

On the one hand, in the spectrum on the left (**6b**), it can be observed a first-order NMR pattern as the signals reproduce an AMX spin system. The α-carbonyl proton X appears at 3.93 ppm and gives rise to a doublet of doublets (dd; J = 9.1, J' = 3.7 Hz) due to the different coupling constant with each of the diastereotopic protons of the benzylic CH_2 (3.08 and 2.78 ppm).
On the other hand, an ABX spin system can be noticed in the spectrum on the right (**6b'**). In this case, the α-carbonyl proton X appears at 3.60 ppm and gives rise to a triplet (t; J = 5.6 Hz) due to the very similar coupling constant with each of the diastereotopic benzylic protons (multiplet centered at 3.11 ppm). In fact, the signals can be easily mistaken for an A_2X spin system. This peculiar phenomenon could be caused by a high degree of rotational freedom of the benzyl group in this isomer.
We hypothesized that a possible explanation for different behaviour exhibited by the two diastereomers in their ^{1}H NMR spectra would be that in the *cis* isomer the phenyl group experiences an attractive π-stacking interaction with the porphyrin moiety, that strongly restricts the conformational mobility of the benzyl substituent. This interaction is no longer possible in the *trans* isomer, that should therefore exhibit a much higher degree of conformational mobility for the same substituent (Figure S2).
In addition to the aromatic stacking, the constricted rotation of the benzyl group in the *cis* isomer could be also caused by a significant destabilizing steric interaction between the benzyl substituent and the adjacent *meso*-phenyl rings, which are arranged nearly perpendicular to the porphine core (Figure S2). In the same way, the absence of this steric congestion in the *trans* isomer would enhance the rotational freedom of the benzyl substituent.

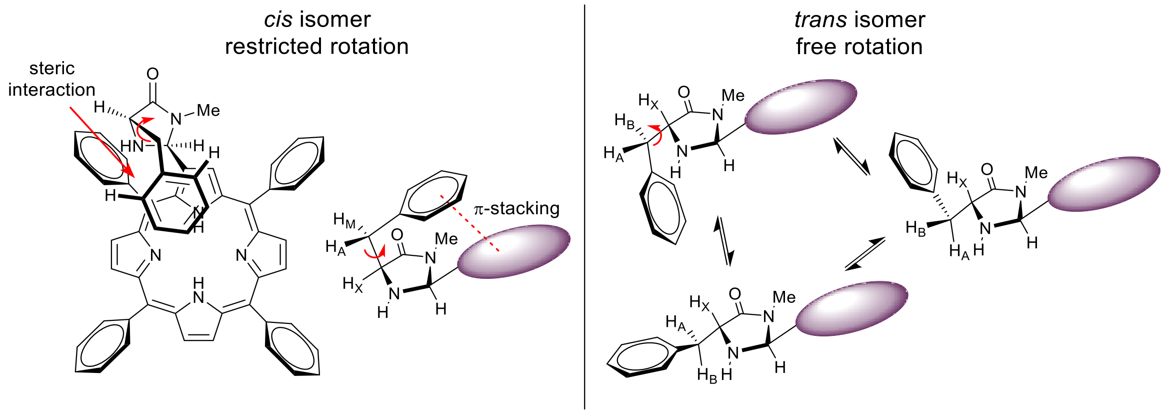

Figure S2. Comparison of the rotational freedom of the benzyl group in the *cis* and *trans* isomers.

In order to understand the different behaviour exhibited by the two diastereomers in their ^{1}H NMR spectra, we decided to evaluate the conformer distribution of both *cis* and *trans* isomers. We were

pleased to find that molecular mechanics calculations gave support to our hypothesis. The conformer distribution calculations were performed using Spartan 14 employing Monte-Carlo with the MMFF force field. Redundant conformers were manually removed.

A simple analysis of the three lowest energy conformations (Figure S3) for each isomer indicates that the benzyl group of the *cis* isomer seems to be able to interact with the π-electrons of the porphyrin ring, which could lead to some sort of π-stacking interaction that could constrain the geometry of this isomer. The benzyl ring of the *trans* isomer, on the other hand, does not show any appreciable aromatic interaction with the porphyrin moiety and would be less sterically compromised for free rotation.

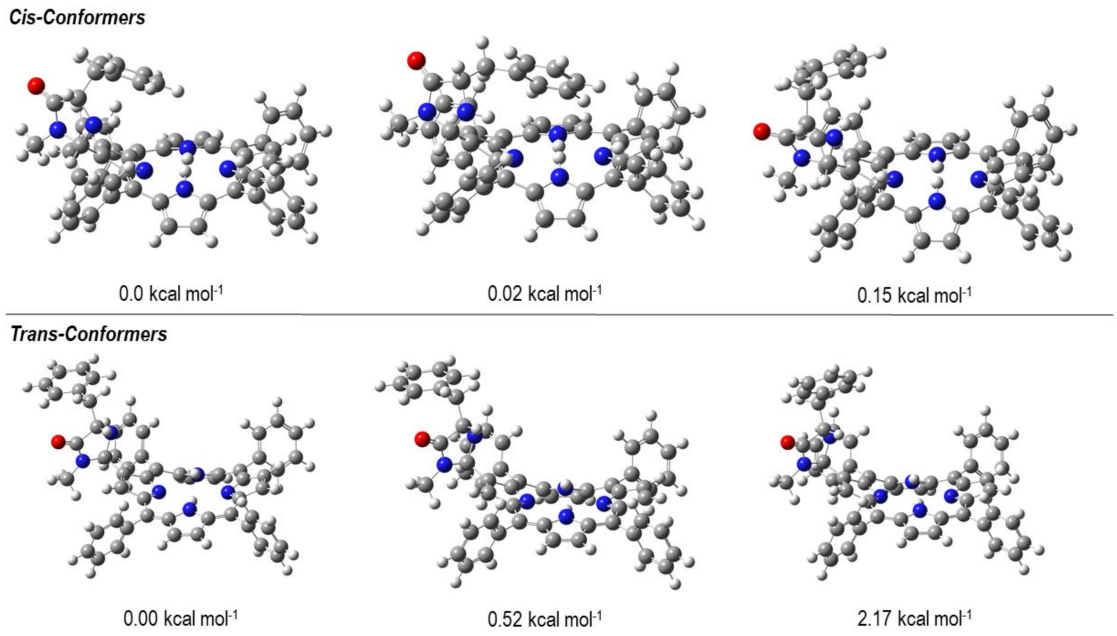

Figure S3. Three lowest energy conformers for the *cis* (top) and *trans* (bottom) isomers and their relative energies (MMFF)

Accordingly, we could conclude that the isomer **6b**, exhibiting an AMX subspectrum, with different chemical shifts for the two methylene protons and also with different J_{AX} and J_{MX} coupling constants should therefore correspond to the *cis* isomer (Figure S1, left). Contrarywise, for the *trans* isomer **6b’** the rapid interconversion between the conformers with similar stabilities would result in the averaging of both the chemical shifts and of the coupling constants, leading to an ABX subspectrum in which $J_{AX} = J_{BX}$ (Figure S1, right).

Even though the calculations were only performed at the molecular mechanics level of theory, they give an idea of which NMR spectrum corresponds to which isomer. ^{1}H-^{1}H NOESY NMR and ^{1}H-^{1}H ROESY NMR spectra of both diastereomers were performed to evaluate the spatial proximity of the hydrogens of the imidazolidinone ring of the *cis* isomer. Unfortunately, we were unable to detect any diagnostic signals to confirm our assignment.

Experimental procedures for the preparation of compounds $TPPH_2$, $Na_4TPPH_2S_4$, $(H_3O)_2TPPH_4S_4$, 4a-c, 18, 25, 32, 33, and 34

5,10,15,20-Tetraphenylporphyrin ($TPPH_2$). A 500 mL round-bottomed flask, equipped with magnetic stirring and a Liebig reflux condenser was filled in with 300 mL of propionic acid and heated until reflux. At this point, freshly distilled pyrrole (5.4 mL, 77.6 mmol) and benzaldehyde (7.92 mL, 77.8 mmol) were added simultaneously through the condenser, so that any momentary excess of one of the reactants in the reaction mixture is avoided. The reaction mixture was stirred for 30 min under reflux and cooled down to room temperature. Then, the resulting suspension was filtered in a Büchner funnel and the collected solid was washed with MeOH until the washings were colorless. The solid was dried under vacuum in the desiccator to obtain the porphyrin as a purple crystalline solid (2.52 g, 21% yield).

^{1}H-NMR ($CDCl_3$, 400 MHz): δ = 8.85 (s, 8H), 8.25-8.20 (dd, J = 7.5 Hz, J' = 1.5 Hz, 8H), 7.82-7.72 (m, 12H), -2.77 (br, 2H) ppm. **UV-Vis** [DCM, λ_{max} (ε), c = 2.05 x 10^{-6} M]: 417 (488000), 517 (16000), 549 (9600), 591 (6900), 649 (4600) nm. [52]

5,10,15,20-Tetrakis(4-sulfonatophenyl)porphyrin tetrasodium salt ($Na_4TPPH_2S_4$). In a 100 mL round-bottomed flask, equipped with magnetic stirring and a Dimroth reflux condenser, **$TPPH_2$** (1.00 g, 1.6 mmol) and concentrated H_2SO_4 (98 %, 55 mL) were added sequentially, and the resulting mixture was stirred and heated up to 100 ºC. Stirring at 100 ºC was maintained for 6 h, the mixture was then cooled down to room temperature and stirred overnight. At this point, the resulting green suspension was carefully poured over H_2O MilliQ (15 mL), was distributed in 25 mL vials, and centrifuged to 6000 rpm for 40 minutes to obtain the porphyrin's J-aggregate as a dark green precipitate; the supernatant liquid was removed by decantation and the remaining solid was dispersed in a small amount of water. The suspension was carefully basified by the portionwise addition of solid Na_2CO_3 until the solution changed its color from green to red. At this point, the solvent was removed under vacuum and the resulting purple solid was dissolved in the minimum amount of MeOH, heated up to reflux and allowed to cool down to room temperature to precipitate the inorganic salts. This process was repeated twice. Then, the solvent was removed under vacuum and the resulting solid was, first, redissolved in water and then lyophilized for 2 days, affording the desired porphyrin sodium salt **$Na_4TPPH_2S_4$** as a purple solid (1.33 g, 88% yield). The purity was then determined by UV-Vis.

^{1}H-NMR (DMSO-d_6, 400 MHz): δ = 8.86 (s, 8H), 8.19 (d, J = 8.1 Hz, 8H), 8.06 (d, J = 8.0 Hz, 8H), -2.96 (br, 2H) ppm. [25]

5,10,15,20-Tetrakis(4-sulfophenyl)porphyrin ($(H_3O)_2TPPH_4S_4$). In a 100 mL round-bottomed flask, equipped with magnetic stirring and a Dimroth reflux condenser, **$TPPH_2$** (500 mg, 0.8 mmol) and concentrated H_2SO_4 (98 %, 7 mL) were added sequentially, and the resulting mixture was stirred and heated up to 100 ºC. Stirring at 100 ºC was maintained for 6 h, the mixture was then cooled down to room temperature and stirred overnight. At this point, the resulting green suspension was carefully poured over H_2O MilliQ (15 mL), was distributed in 25 mL vials, and centrifuged to 6000 rpm for 40 minutes to obtain the porphyrin's J-aggregate as a dark green precipitate; the supernatant was carefully separated and the precipitate was washed again with 15 mL more of H_2O MilliQ. This process was repeated until the pH of the supernatant liquid was constant, with a value close to 1.5. Then, the resulting solid was washed with an aqueous solution

of 0.1M HCl and two more times with H_2O MilliQ. Finally, the product was lyophilized for 2 days, affording the desired porphyrin in its zwitterionic form (**$(H_3O)_2TPPH_4S_4$**) as a green solid (480 mg, 63% yield).

^{1}H-NMR (DMSO-d_6, 400 MHz): δ = 8.79 (s, 8H), 8.69 (d, J = 8.0 Hz, 8H), 8.32 (d, J = 8.1 Hz, 8H) ppm. [25]

L-Phenylalanine methyl ester hydrochloride. In a 100 mL round-bottomed flask, equipped with magnetic stirring, 25 mL of absolute MeOH were introduced and cooled down to -10 oC (salt water/ice bath). At this point, $SOCl_2$ (7 mL, 96 mmol) was added via syringe and the reaction mixture was stirred for 10 min at the same temperature. Then, L-phenylalanine (1.65 g, 10.0 mmol) was added, and the solution was stirred for 40 additional minutes at -10 oC and 48 h at room temperature. Next, the reaction mixture was concentrated under reduced pressure, 15 mL of MeOH were added to the residue, and the resulting solution was concentrated under reduced pressure. Addition of MeOH (15 mL) and concentration under vacuum were repeated, 50 mL of diethyl ether were added, and the resulting precipitate was filtered and recrystallized from MeOH/Et_2O to afford the methyl ester hydrochloride as a colorless solid (1.97 g, 88% yield), that was subsequently used without further purification. [42]

L-*tert*-Leucine methyl ester hydrochloride. In a 25 mL round-bottomed flask, equipped with magnetic stirring and a Dimroth reflux condenser, L-*tert*-leucine (760 mg, 5.9 mmol) was dissolved in absolute MeOH (6 mL) and the reaction was cooled down to -10 oC (salt water/ice bath). At this point, SOCl2 (0.90 mL, 12.4 mmol) was added dropwise, and the mixture was stirred for 10 min at -10 oC and for 6 h under reflux. After this time, the mixture was allowed to cool down to room temperature and concentrated under reduced pressure to give a solid, that was mixed with 10 mL of toluene and again removed under reduced pressure in order to remove traces of water that could remain in the reaction crude. This process was repeated three times before drying the crude product under vacuum overnight to afford the methyl ester hydrochloride (1.03 g, quantitative yield) as a yellowish solid, that was subsequently used without further purification. [43]

General procedure for the obtention of *N*-Methyl-α-aminoamide hydrochlorides. In a 10 mL round-bottomed flask, equipped with magnetic stirring, the suitable α-aminoacid methyl esther hydrochloride was added to an aqueous solution of methylamine (2.0 mL, 40 wt. %, 23.1 mmol) and the resulting mixture was stirred at room temperature for 20 h. Then, absolute ethanol (20 mL) was added, and the solution was concentrated under reduced pressure. This process was repeated three times in order to remove the excess of methylamine and water. Finally in order to remove traces of water that could remain in the crude, the solid was mixed with toluene and evaporated under vacuum, affording the corresponding methylamide hydrochloride as a colorless solid, that was subsequently used without further purification.

L-Alanine methylamide hydrochloride. Commercial L-alanine methyl ester hydrochloride (16.2 mmol) was used in the reaction. White solid, 1.97 g, 88% yield.

L-Phenylalanine methylamide hydrochloride. Yellowish solid, 1.30 g, quantitative yield (from 5.80 mmol of L-phenylalanine methyl ester hydrochloride). [41]

L-*tert*-Leucine methylamide hydrochloride. In a thick-wall glass reaction tube equipped with magnetic stirring and a screw top, L-*tert*- leucine methyl ester hydrochloride (1.03 g, 5.7 mmol) was added to a solution of methylamine in EtOH (7.00 mL, 33% wt. , 54.5 mmol). Then, the reaction tube was tightly sealed, and heated up to 100 ºC for 48 h, and subsequently stirred for 3 days at room temperature. At this point, the solution was transferred to a round-bottomed flask and concentrated under reduced pressure to afford the desired methylamide hydrochloride as a yellowish solid (1.00 g, quantitative yield), that was not characterized and was used without further purification in subsequent reactions. [43]

General procedure for the obtention of *N*-Methyl-α-aminoamides. In a 10 mL round-bottomed flask, equipped with magnetic stirring and a Dimroth reflux condenser, the suitable α-aminoacid methylamide hydrochloride was added to a 1 M solution of NaOH in EtOH (3 mL) and heated up to reflux for 2 min. Then, a digestion of the crude was carried out with EtOAc to insolubilize the formed NaCl. At this point, the suspension was filtered through a cotton plug in a Pasteur pipette and evaporated *in vacuo* to afford the desired α-aminoacid methylamide, that was not characterized and was directly used in the next reaction without further purification.

L-Alanine methylamide (4a). Pale orange solid, 408 mg, quantitative yield (from 1.80 mmol of the L-alanine methylamide hydrochloride).

L-Phenylalanine methylamide (4b). Yellowish oil, 370 mg, quantitative yield (from 2.27 mmol of the L-phenylalanine methylamide hydrochloride). [41]

L-*tert*-Leucine methylamide (4c). In a 10 mL round-bottomed flask, equipped with a magnetic stirrer, L-*tert*-leucine methylamide hydrochloride (285 mg, 1.6 mmol) was dissolved in a mixture of water (3 mL) and concentrated NH_3 (0.15 mL, 2.5 mmol). Once the solid was completely dissolved, the solution was extracted with a 20 % mixture of 2-propanol in DCM (5 x 20 mL). Then, the organic phase was dried over $MgSO_4$ and concentrated under reduced pressure to give a solid, that was mixed with 10 mL of toluene and again evaporated under reduced pressure in order to remove traces of water that could remain in the reaction crude. This process was repeated three times before drying the crude reaction product to afford an orange solid (123 mg, 54% yield), that was not characterized and was directly used in next reaction without further purification. [43]

(*S*)-Prolinol (14). In a 500 mL round-bottomed flask, equipped with magnetic stirring and a reflux condenser, 200 mL of anhydrous commercial THF were introduced. Then, $LiAlH_4$ (6.89 g, 181.5 mmol) was carefully added, followed by the portion wise addition of L-proline (6.97g, 60.5 mmol), noticing intense bubbling. The resulting grey suspension was vigorously stirred at room temperature for 24 h. At this point, 10 mL of H_2O were carefully added to the reaction mixture, followed by the slow sequential addition of 10 mL of a 15 % (w/v) NaOH aqueous solution and by 10 mL of H_2O. At this point, the precipitate color change from grey to white was observed. Once the color change was complete, 200 mL of diethyl ether were added, and the resulting white crystalline solid was filtered out and washed with 3 x 50 mL of ethyl acetate. The combined organic layers were concentrated under vacuum to afford a yellowish oil (4.38 g, 72% yield), that was directly used in the next step without further purification.

^{1}H-NMR ($CDCl_3$, 400 MHz): δ = 3.53 (q, *J* = 6.6 Hz, 1H), 3.35-3.32 (m, 2H), 2.96-2.76 (m + br, 4H), 1.87-1.67 (m, 3H), 1.45-1.37 (m, 1H) ppm. [46]

***N*-Boc-(*S*)-prolinol (15).** To a magnetically stirred solution of (*S*)-Prolinol **14** (4.38 g, 43.4 mmol) in DCM (125 mL), triethylamine (21.7 mL, 156.1 mmol) was added, followed by di(*tert*-butyl)dicarbonate (11.36 g, 52.0 mmol). The mixture was stirred for 2 h at room temperature. At this point, 50 mL of H_2O were added, and the two layers were separated. The organic phase was washed with 2 x 20 mL of H_2O, dried over anhydrous Na_2SO_4 and concentrated under vacuum to afford a red oil. The crude product was purified by flash column chromatography through deactivated silica gel (2.5% v/v NEt_3) and using an eluent gradient from hexane/AcOEt (5/1) to hexane/AcOEt (1/1), to give the protected prolinol **15** as a yellowish oil (3.65 g, 72% yield).

^{1}H-NMR ($CDCl_3$, 400 MHz): δ = 4.75 (br, 1H), 3.98-3.96 (m, 1H), 3.66- 3.55 (m, 2H), 3.48-3.42 (m, 1H), 3.34-3.28 (m, 1H), 2.01 (sext, *J* = 5.5 Hz, 1H), 1.81 (m, 3H), 1.47 (s, 9H) ppm. [46]

***N*-Boc-(*S*)-2-(4-toluenesulfonyloxy)-methyl pyrrolidine (16).** To an ice-cold solution of *N*-Boc-L-prolinol **15** (2.93 g, 14.6 mmol) in pyridine (23 mL), placed in a 250 mL round-bottomed flask equipped with magnetic stirring, 4-toluenesulfonyl chloride (4.70 g, 24.7 mmol) was added and the resulting mixture was stirred for 6 h at room temperature. At this point, Et_2O (200 mL) was added and the two-phase mixture was transferred to a separatory funnel. The organic phase was washed successively with HCl 10% (2 x 50 mL), a saturated aqueous solution of $NaHCO_3$ (3 x 50 mL) and brine (3 x 50 mL), dried over Na_2SO_4 and concentrated under reduced pressure. The resulting orange solid was purified via flash column chromatography through silica gel using Hex/AcOEt (3/2) as eluent, affording the desired product **16** (2.91 g, 56% yield) as a yellowish oil.

^{1}H-NMR ($CDCl_3$, 400 MHz): δ = 7.76 (d, *J* = 8.2 Hz, 2H), 7.73 (d, *J* = 8.2 Hz, 2H), 3.95-3.90 (m, 2H), 3.30-3.25 (m, 3H), 2.44 (s, 3H), 1.86-1.80 (m, 4H), 1.47 (s, 9H) ppm. [46]

***N*-Boc-(*S*)-2-azidomethylpyrrolidine (17).** In a 250 mL round bottomed flask, equipped with magnetic stirring and a Dimroth reflux condenser, *N*-Boc-(*S*)-2-(4-toluensulfonyloxy)-methylpyrrolidine **16** (2.914 g, 8.2 mmol) was dissolved in anhydrous DMSO (87 mL). Then, sodium azide (3.195 g, 49.1 mmol) was added and the mixture was stirred for 19 h at 64 ºC. At this point, it was allowed to cool down until room temperature, diluted with Et_2O (166 mL) and transferred to a separatory funnel. The organic phase was washed with water (3 x 120 mL) and brine (1 x 60 mL), dried with Na_2SO_4 and concentrated under reduced pressure. The desired product **17** (1.556 g, 84% yield) was obtained as a yellowish oil, that did not require further purification.

^{1}H-NMR ($CDCl_3$, 400 MHz): δ = 4.00-3.80 (m, 1H), 3.37 (m, 4H), 1.88-1.82 (m, 4H), 1.47 (s, 9H) ppm. [46]

***tert*-Butyl (*S*)-2-aminomethylpyrrolidine-1-carboxylate (18).** In a 250 mL round bottomed flask, equipped with magnetic stirring and a Dimroth reflux condenser, *N*-Boc-(*S*)-2-azidomethylpyrrolidine **17** (1.556 g, 6.9 mmol) was dissolved in THF (59 mL). Then, triphenylphosphine (3.609 g, 13.8 mmol) and H_2O (0.3 mL) were added and the resulting mixture was heated up to reflux for 24 h. After cooling down to room temperature, the solvents were evaporated under reduced pressure and the resulting oil was redissolved in Et_2O (125 mL); the solution pH was then adjusted to pH = 2 with 1 M aqueous HCl. Then, the aqueous phase was washed with Et_2O (2 x 25 mL), the pH was brought to 12 with aqueous 2 M NaOH and extracted with DCM (6 x 20 mL). The combined organic layers were dried with Na_2SO_4 and concentrated

under reduced pressure to afford **18** as a yellowish oil (1.070 g, 78% yield), that did not require further purification.

^{1}H-NMR ($CDCl_3$, 400 MHz): δ = 3.77-3.73 (m, 2H), 3.28-3.24 (m, 2H), 2.78-2.76 (m, 1H), 2.64-2.60 (m, 1H), 1.81.70 (m, 3H), 1.49 (br, 2H), 1.40 (s, 9H) ppm. [46]

***N*-Boc-L-proline (25).** In a 50 mL round-bottomed flask, equipped with magnetic stirring, L-proline (1.00 g, 8.7 mmol) was dissolved in an aqueous saturated solution of $NaHCO_3$ (13 mL), and cooled down to 0 ºC with an ice-water bath. Then, di-*tert*-butyl dicarbonate (2.09 g, 9.6 mmol) was dissolved in THF (5 mL) and added dropwise to the cold solution. The resulting mixture was stirred for 19 h at room temperature. At this point, THF was removed under reduced pressure and, after cooling down to 0 ºC, the aqueous solution was acidified to pH = 2 with 3 M aqueous HCl (2.7 + 13.3 mL). The aqueous phase was extracted with AcOEt (3 x 15 mL) and the combined organic layers were dried over Na_2SO_4 and concentrated under reduced pressure, to afford the desired product (1.87 g, quantitative yield) as a white solid, that was not characterized and was subsequently used without further purification. [48]

(*S*)-5-Benzyl-2,2,3-trimethylimidazolidin-4-one (32) A 10 mL round-bottomed flask, equipped with magnetic stirring and containing a solution of L- phenylalanine methylamide **4b** (160 mg, 0.9 mmol) in anhydrous MeOH (4.8 mL), was purged with Ar. Then, acetone (0.35 mL, 4.70 mmol) was added and the mixture was heated up to reflux overnight. At this point, the reaction was allowed to cool down to room temperature and concentrated under reduced pressure to afford the desired product **32** (189 mg, 90% yield) as a yellowish oil.

^{1}H-NMR ($CDCl_3$, 400 MHz): δ = 7.35-7.20 (m, 5H), 3.80 (dd, *J* = 6.9 Hz, *J*’ = 4.7 Hz, 1H), 3.15 (dd, *J* = 14.1 Hz, *J’* = 4.7 Hz, 1H), 3.01 (dd, *J* = 14.1 Hz, *J’* = 6.9 Hz, 1H), 2.76 (s, 3H), 1.78 (br, 1H), 1.26 (s, 3H), 1.16 (s, 3H) ppm.

(*S*)-2,2,3,5-Tetramethylimidazolidin-4-one (33). A 10 mL round-bottomed flask, equipped with magnetic stirring and containing a solution of L-alanine methylamide **4a** (200 mg, 2.0 mmol) in anhydrous MeOH (6 mL), was purged with Ar. Then, acetone (0.75 mL, 10.2 mmol) was added and the mixture was heated up to reflux overnight. At this point, the reaction was allowed to cool down to room temperature and concentrated under reduced pressure to afford the desired product **33** (220 mg, 79% yield) as an off-white solid.

^{1}H-NMR ($CDCl_3$, 400 MHz): δ = 3.67 (q, *J* = 6.9 Hz, 1H), 2.80 (s, 3H), 2.06 4 (br, 1H), 1.51 (s, 3H), 1.43 (d, *J* = 6.9 Hz, 3H), 1.39 (s, 3H) ppm. [50]

(2*S*,5*S*)-5-Benzyl-2-*tert*-butyl-3-methylimidazolidin-4-one (34). In a 50 mL round-bottomed flask, equipped with magnetic stirring and Dimroth reflux condenser, and containing L-phenylalanine methylamide **4b** (1.05 g, 5.6 mmol), $CHCl_3$ (15 mL) was added. Once the oil was completely dissolved, pivaldehyde (1.30 mL, 1.03 g, 12 mmol) and $(CF_3SO_2)_3Yb$ (72 mg, 0.12 mmol, 20 mol%) were added sequentially, and the mixture was heated up to reflux overnight. After cooling to room temperature, the reaction mixture was evaporated under reduced pressure and the residue was purified via flash column chromatography through silica gel, using hexane/AcOEt (1/3) as eluent, to afford the desired product **34** (157 mg, 15% yield) as a colorless solid.

^{1}**H-NMR** ($CDCl_3$, 400 MHz): δ = 7.29-7.13 (m, 5H), 4.02 (s, 1H), 3.67 (dd, *J* = 7.4 Hz, *J'* = 3.6 Hz, 1H), 3.12 (dd, *J* = 13.3 Hz, *J'* = 3.6 Hz, 1H), 2.93 6 (dd, *J* = 13.3 Hz, *J'* = 7.4 Hz, 1H), 2.88 (s, 3H), 2.01 (br, 1H), 0.80 (s, 9H) ppm. [43]

NMR spectra of new compounds

2-((2*R*,4*S*)-1,4-*D*imethyl-5-oxoimidazolidin-2-yl)-5,10,15,20-tetraphenylporphyrin (6a)

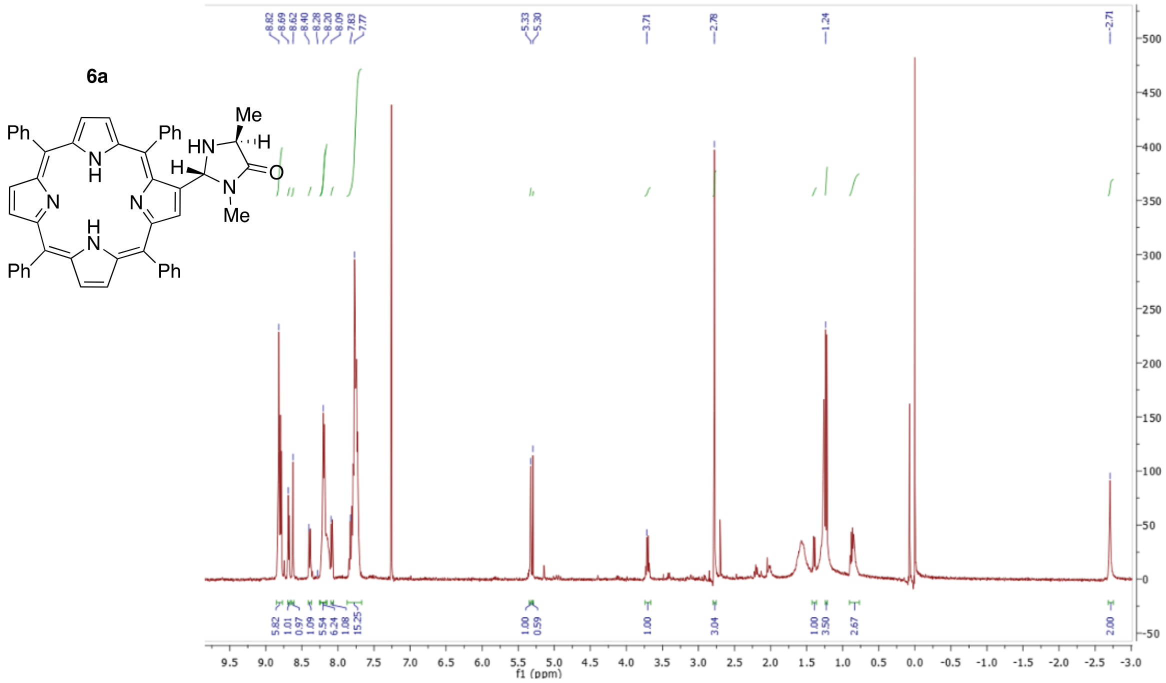

^{1}H-NMR (400 MHz, $CDCl_3$, TMS_{int})

Figure S4

2-((2*S*,4*S*)-4-Benzyl-1-methyl-5-oxoimidazolidin-2-yl)-5,10,15,20-tetraphenylporphyrin (6b-*cis*)

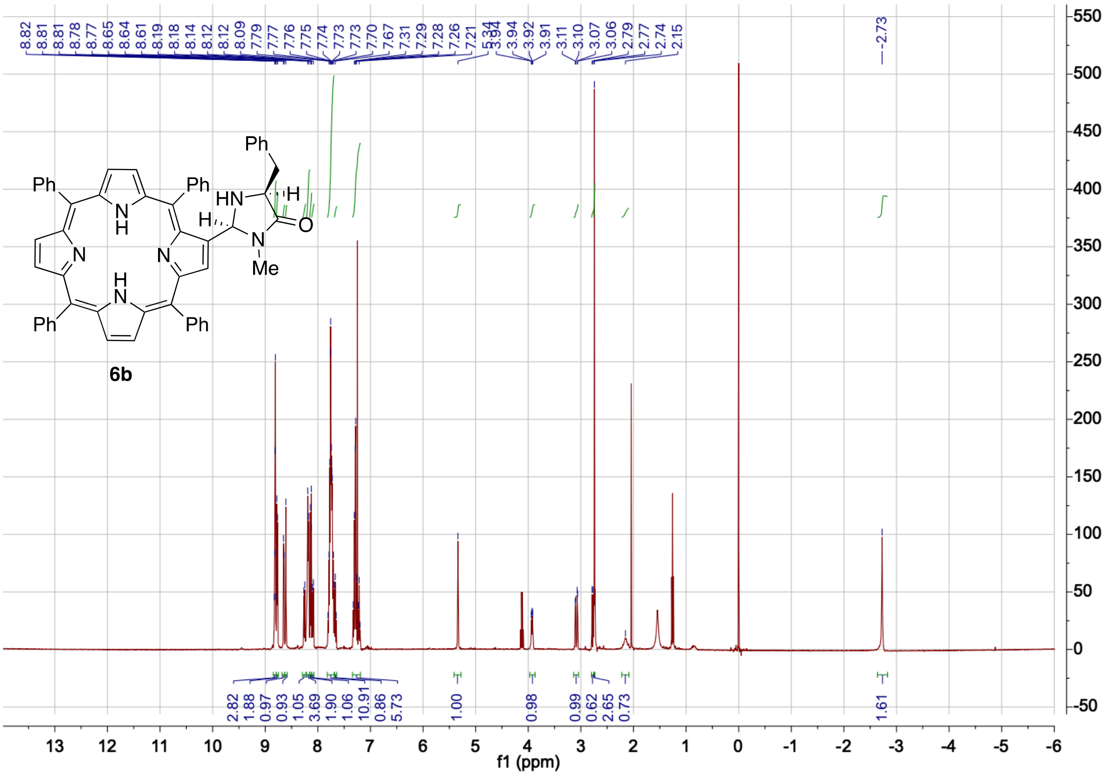

^{1}H-NMR (400 MHz, $CDCl_3$, TMS_{int})

Figure S5

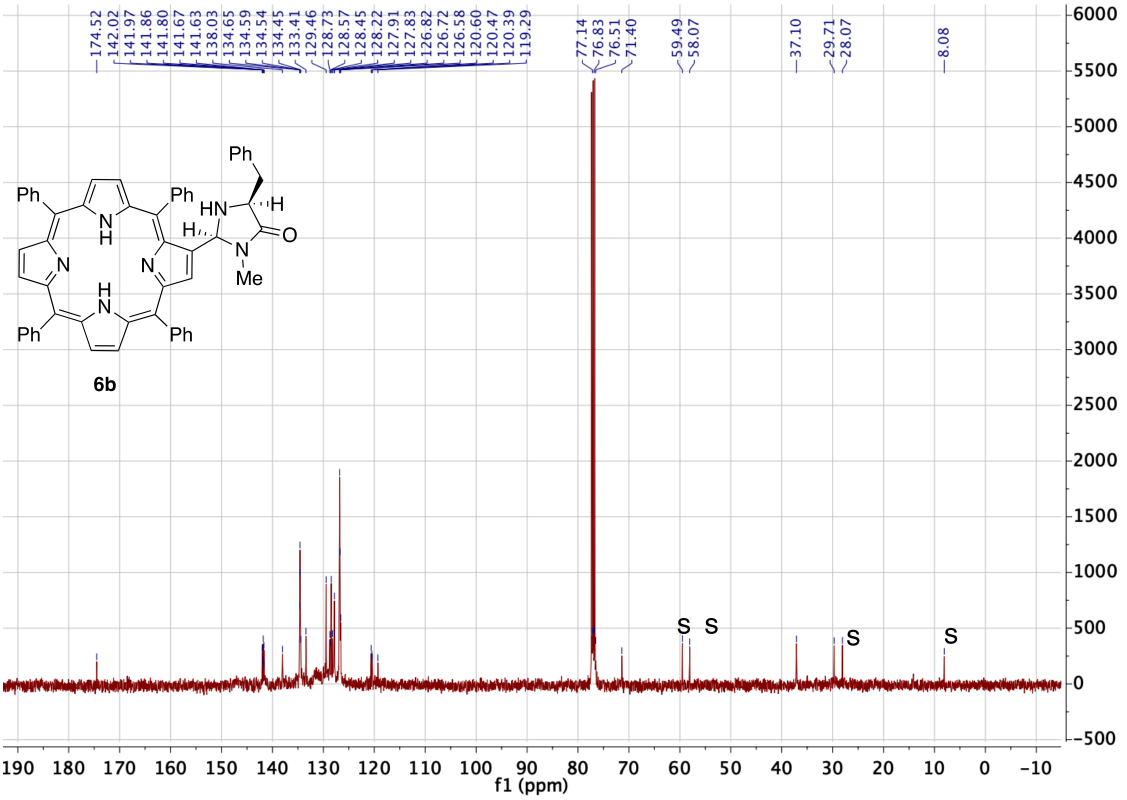

^{13}C-NMR (100 MHz, $CDCl_3$)

Figure S6

2-((2*R*,4*S*)-4-Benzyl-1-methyl-5-oxoimidazolidin-2-yl)-5,10,15,20-tetraphenylporphyrin (6b'-*trans*)

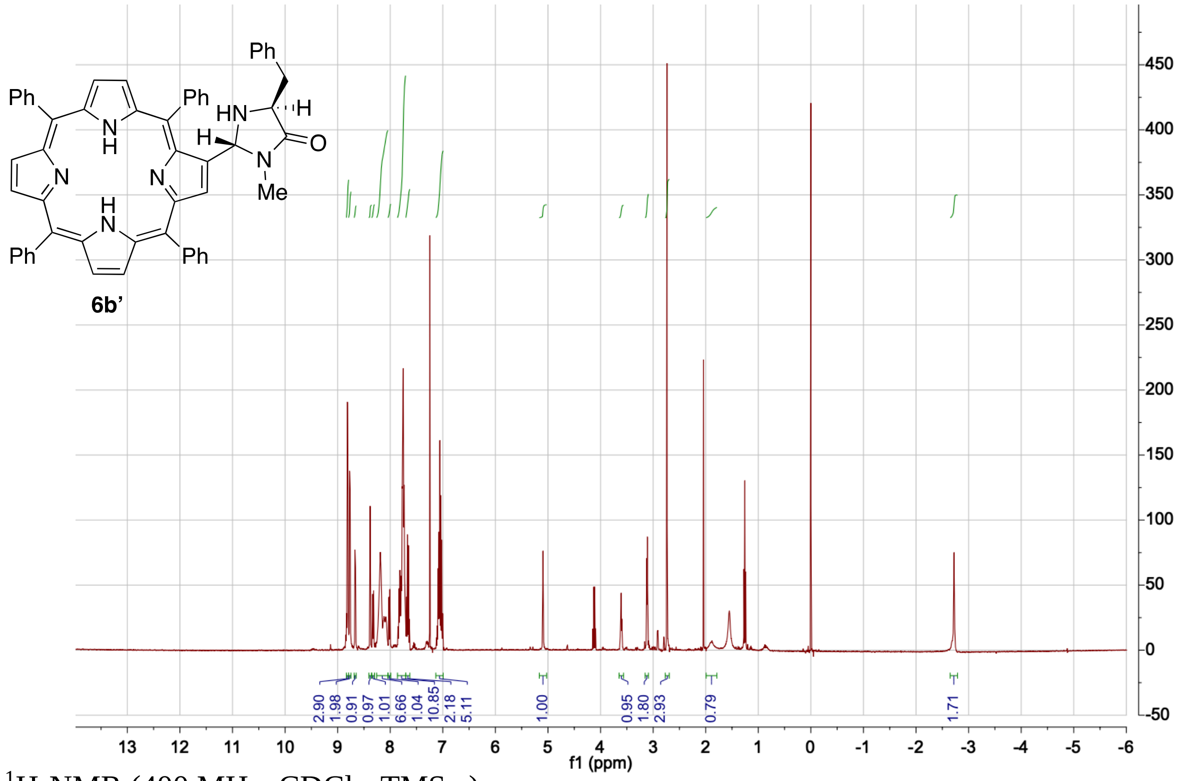

^{1}H-NMR (400 MHz, $CDCl_3$, TMS_{int})

Figure S7

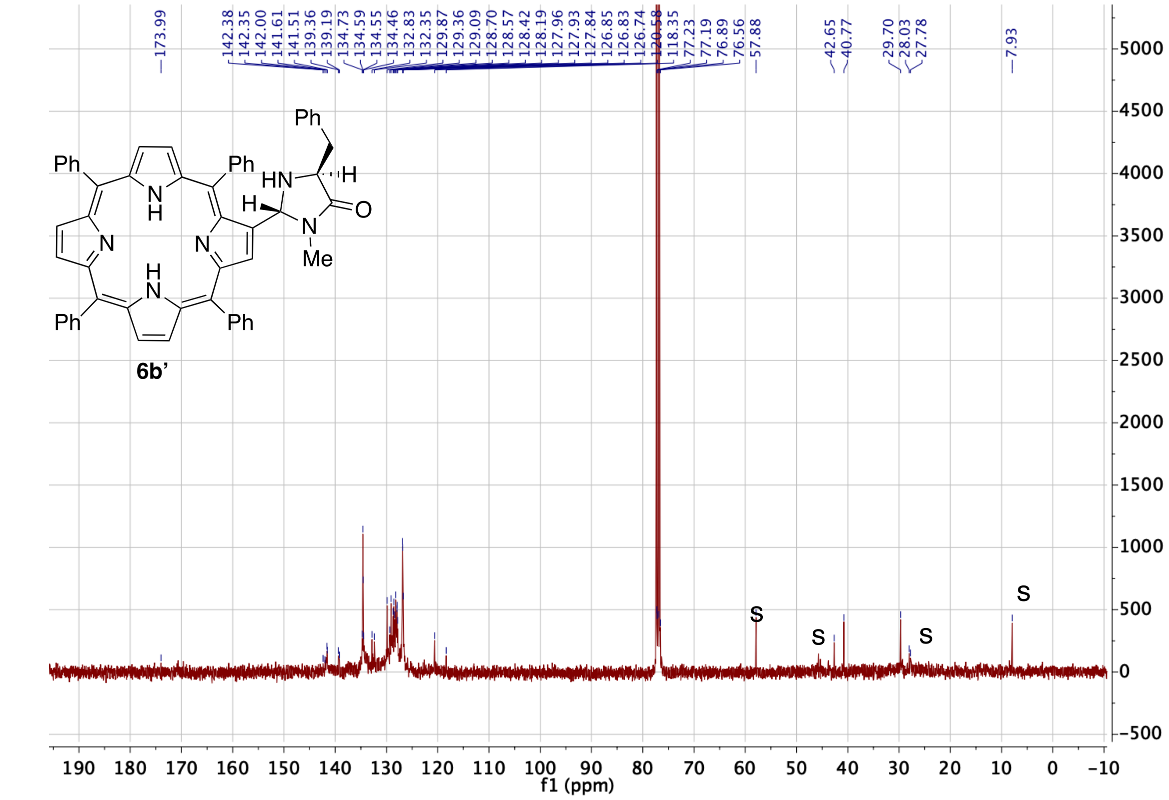

^{13}C-NMR (100 MHz, $CDCl_3$)

Figure S8

2-((2*R*,4*S*)-4-(*tert*-Butyl)-1-methyl-5-oxoimidazolidin-2-yl)-5,10,15,20-tetraphenylporphyrin (6c)

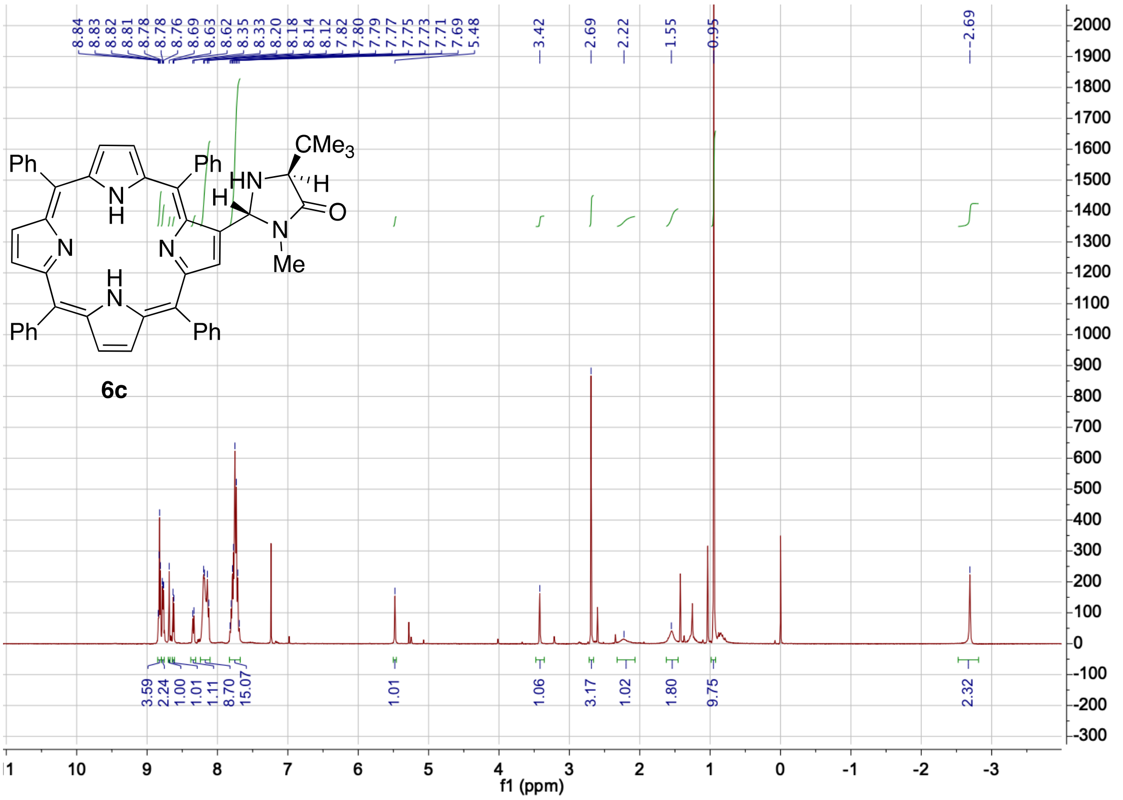

^{1}H-NMR (400 MHz, $CDCl_3$, TMS_{int})

Figure S9

(*S*)-5-(4-((((1-(*tert*-Butoxycarbonyl)pyrrolidin-2-yl)methyl)amino)methyl)phenyl)-10,15,20-triphenylporphyrin (19)

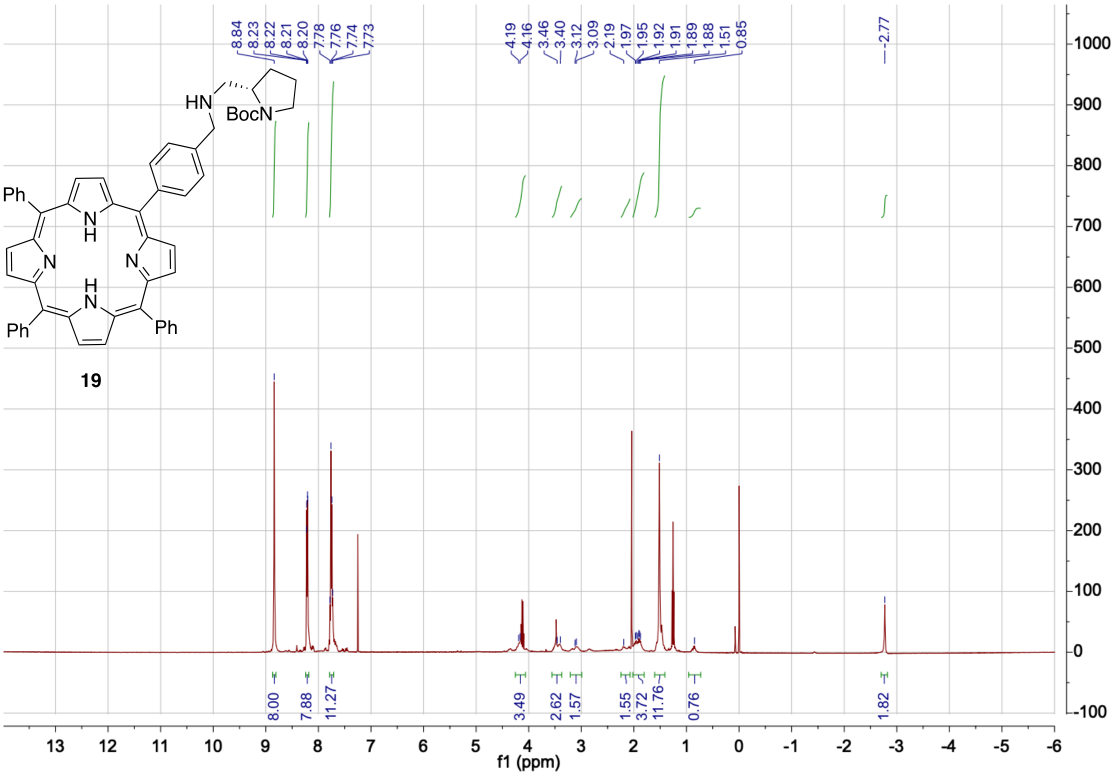

^{1}H-NMR (400 MHz, $CDCl_3$, TMS_{int})

Figure S10

(*S*)-5,10,15-Triphenyl-20-(4-(((2-(pyrrolidin-2-yl)ethyl)amino)methyl)phenyl)porphyrin (20)

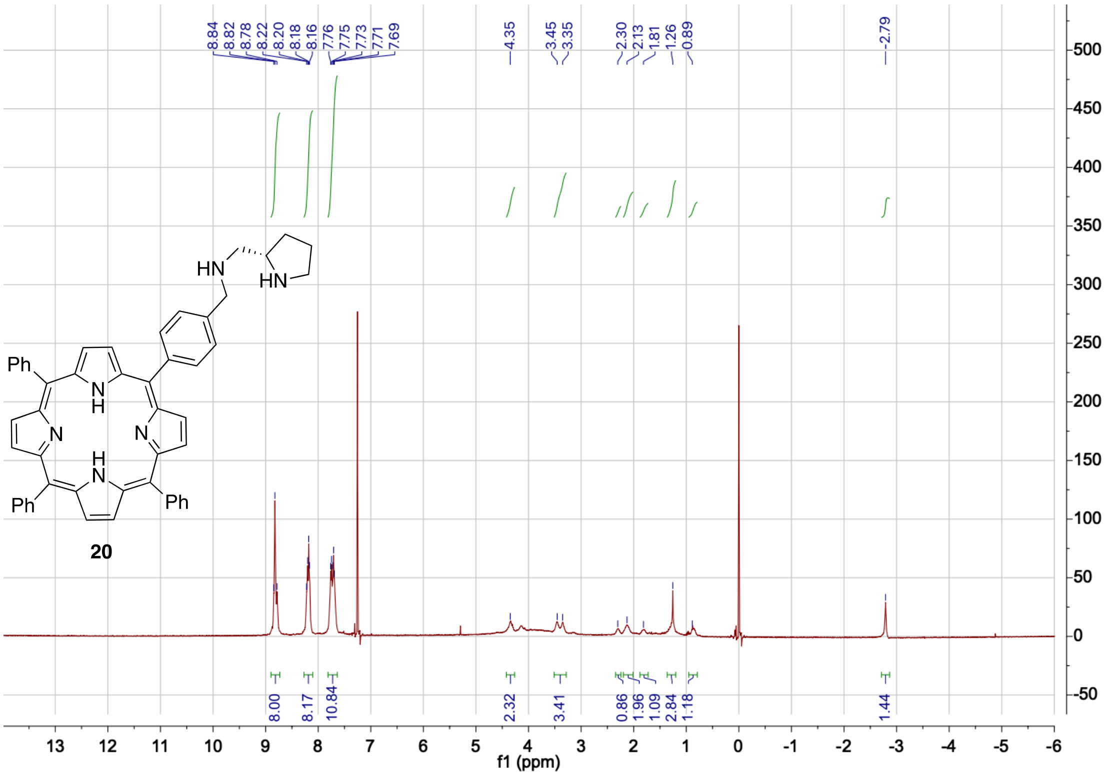

^{1}H-NMR (400 MHz, $CDCl_3$, TMS_{int})

Figure S11

(*S*)-5-(4-((1-(*tert*-Butoxycarbonyl)pyrrolidine-2-carboxamido)methyl)phenyl)-10,15,20-triphenylporphyrin (26)

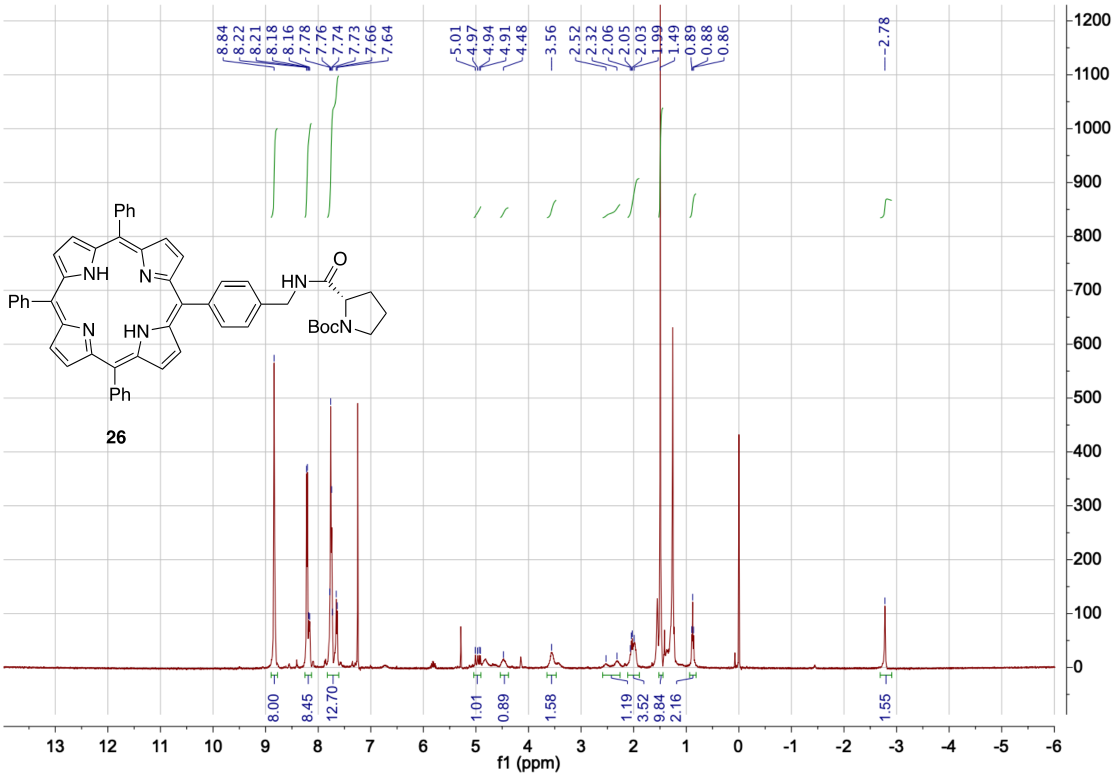

^{1}H-NMR (400 MHz, $CDCl_3$, TMS_{int})

Figure S12

(*S*)-*N*-(4-(10,15,20-Triphenylporphyrin-5-yl)benzyl)pyrrolidine-2-carboxamide (27)

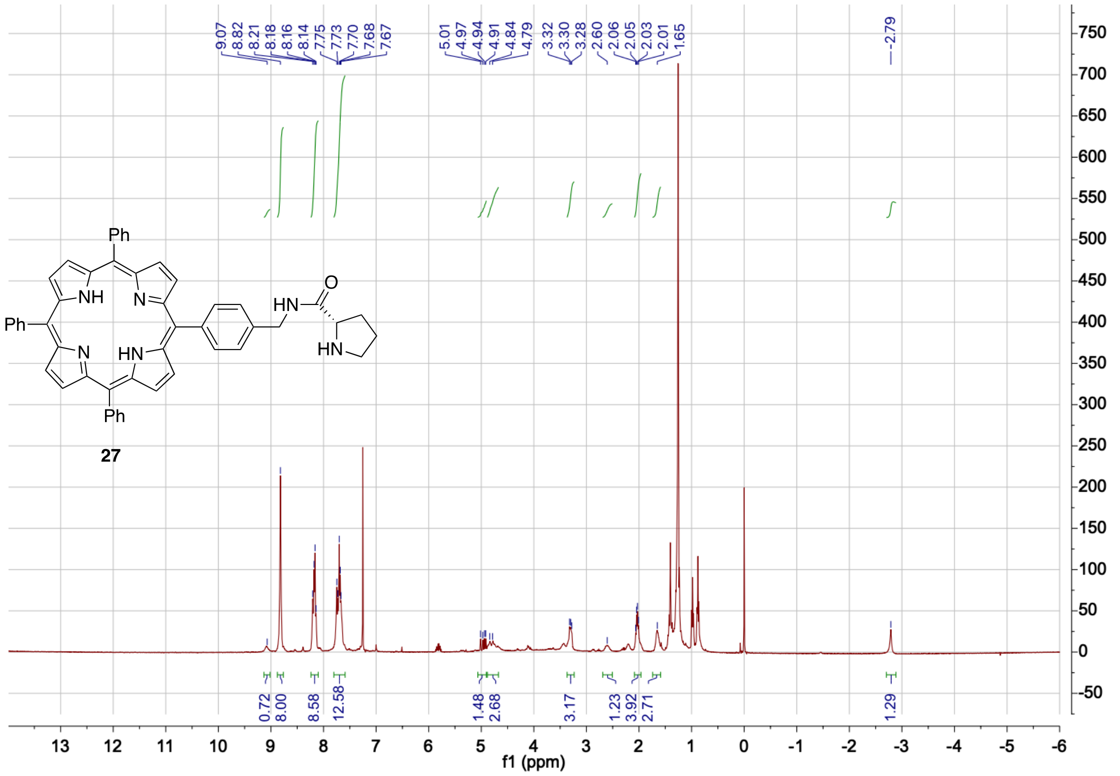

^{1}H-NMR (400 MHz, $CDCl_3$, TMS_{int})

Figure S13

Determination of the stereochemical composition of the products of the Diels–Alder cycloaddition of cyclopentadiene (28) and (*E*)-cinnamaldehyde (29)

The determination of the diastereomeric ratio of the adducts was performed directly on the reaction crudes by ^{1}H-NMR, since the *endo*/*exo* diastereomers exhibit a different chemical shift for the aldehyde proton signal appearing between 9.50 and 10.00 ppm . The *exo* diastereomer **31** gives rise to a doublet at a chemical shift of 9.92 ppm, whereas the *endo* diastereomer **30** exhibits a doublet at 9.60 ppm. The doublet observed at 9.72 ppm corresponds to the unreacted starting aldehyde **29** (Figure S14).

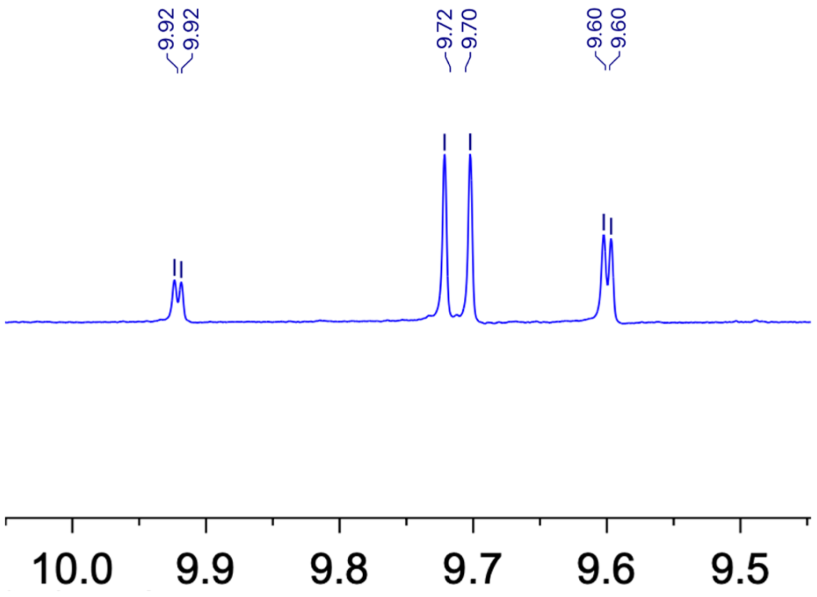

Figure S14

Determination of the **30**/**31**diastereomeric ratio of the Diels-Alder reaction by ^{1}H-NMR ($CDCl_3$, 400 MHz).

The enantiomeric excess of both adducts was determined via HPLC in a chiral stationary phase. For this purpose, since it was highly complicated to find optimal conditions for the separation of the four stereoisomeric aldehydes, we decided to derivatize them *in situ* to the corresponding alcohol mixture. The reduction was carried out with $NaBH_4$ in MeOH.

After this reduction was performed, the enantiomeric excess was determined under the following conditions: Phenomenex i-cellulose 5 column; hexane/IPA 0.8%; flow rate 1 mL/min, λ = 210 nm. The absolute configuration of the products was assigned in accordance with the data reported in the literature [51] (Figure S15).

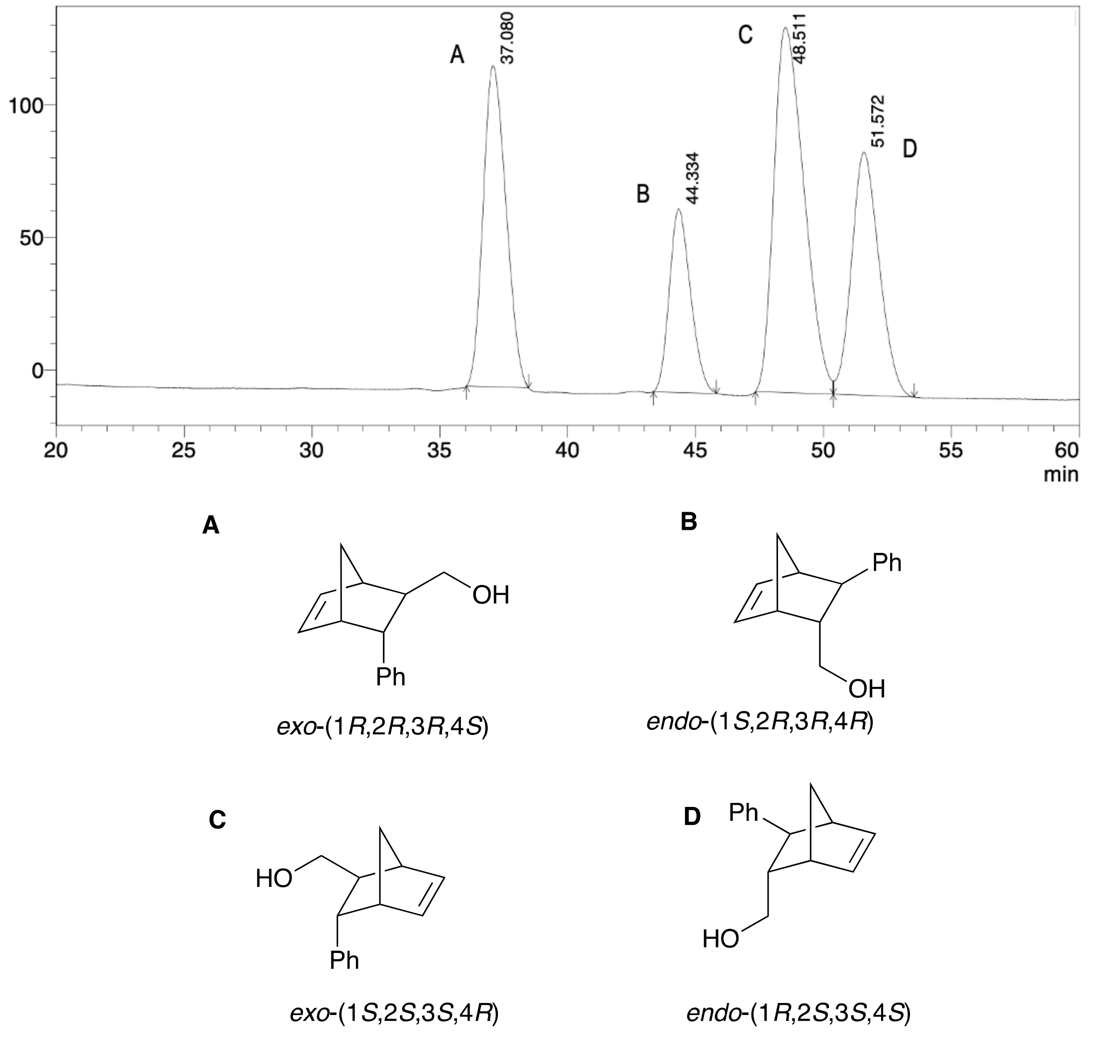

Figure S15

HPLC spectrum from a racemic mixture of the alcohol derivatization products (top). HPLC conditions: Phenomenex i-cellulose 5 column; hexane/IPA 0.8%; flow rate 1 mL/min, λ = 210 nm. Assignment of the absolute configuration of the derivatization products (bottom)
